# Assessing computed tomography image quality for combined detection and estimation tasks

**DOI:** 10.1117/1.JMI.4.4.045503

**Published:** 2017-11-21

**Authors:** Hsin-Wu Tseng, Jiahua Fan, Matthew A. Kupinski

**Affiliations:** a University of Arizona, College of Optical Sciences, Tucson, Arizona, United States; b GE Healthcare, CT Systems Engineering, Waukesha, Wisconsin, United States

**Keywords:** computed tomography, iterative reconstruction, channelized scanning linear observer, detection, estimation, estimation receiver operating characteristic, EROC curves

## Abstract

Maintaining or even improving image quality while lowering patient dose is always the desire in clinical computed tomography (CT) imaging. Iterative reconstruction (IR) algorithms have been designed to allow for a reduced dose while maintaining or even improving an image. However, we have previously shown that the dose-saving capabilities allowed with IR are different for different clinical tasks. The channelized scanning linear observer (CSLO) was applied to study clinical tasks that combine detection and estimation when assessing CT image data. The purpose of this work is to illustrate the importance of task complexity when assessing dose savings and to move toward more realistic tasks when performing these types of studies. Human-observer validation of these methods will take place in a future publication. Low-contrast objects embedded in body-size phantoms were imaged multiple times and reconstructed by filtered back projection (FBP) and an IR algorithm. The task was to detect, localize, and estimate the size and contrast of low-contrast objects in the phantom. Independent signal-present and signal-absent regions of interest cropped from images were channelized by the dense-difference of Gauss channels for CSLO training and testing. Estimation receiver operating characteristic (EROC) curves and the areas under EROC curves (EAUC) were calculated by CSLO as the figure of merit. The one-shot method was used to compute the variance of the EAUC values. Results suggest that the IR algorithm studied in this work could efficiently reduce the dose by ∼50% while maintaining an image quality comparable to conventional FBP reconstruction warranting further investigation using real patient data.

## 1 Introduction

The advent of multiple detector computed tomography has led to various approaches in medical computed tomography (CT) imaging. Its rapid volume acquisition makes CT angiography and CT colonography routine. However, the wide and increasing usage of CT in modern medicine has increased patient dose, which has become a concern in the CT community.^1^^,^^2^ Discussions regarding the associated estimated risk for radiation-induced cancer have been well publicized.^3^^–^^7^ Regardless of the merits of these various arguments, it is always desirable to limit the dose to the patient. Consequently, different hardware and algorithm solutions have been developed to maintain or even improve the image quality while the radiation dose is reduced. The traditional CT reconstruction algorithm filtered back projection (FBP) is well known for its speed and robust image quality. However, FBP images suffer from noise and artifact contaminations especially in low radiation dose conditions. To lower the radiation dose without sacrificing image quality, several iterative reconstruction (IR) algorithms were developed and introduced commercially in the past several years.^8^^–^^11^ The challenge of reducing dose is to maintain a clinically acceptable image quality while decreasing exposure level.

To evaluate the image qualities of different image systems, state-of-the-art medical image quality assessment methods that extract desired information from the images for clinically interesting tasks are now known to be good choices. A clinically interesting and relevant task might be a detection task that requires distinguishing normal cases from diseased cases. Another example of a clinically relevant task is an estimation task where the observer must provide information regarding, for example, the size and contrast of the lesions. Image quality in medical systems should be measured by an observer performing a task or tasks of clinical interest. However, there is not a clear choice for this task in CT imaging. Our previous studies have shown different dose-savings depending upon the choice of task.^12^^,^^13^ In this study, we focused on the application of the channelized scanning linear observer (CSLO) on CT images to quantitatively evaluate the performance of different reconstruction algorithms under tasks that include signal detection, localization, and estimation of size and contrast in an attempt to obtain a more complete picture of dose savings. It should be noted that this paper will focus on a mathematical observer model and that validation of this observer model against human performance will not be discussed in this paper but will be addressed in a future publication. The outline of this paper is as follows: Sec. 2 describes the materials and methods used. More specifically, Sec. 2.1 illustrates the idea of the scanning linear observer (SLO); Sec. 2.2 depicts the selection of channels and the concept of the channelization mechanism applied on the SLO; Sec. 2.3 outlines the training dataset, testing dataset, and the estimation method of variance for observer study; Sec. 2.4 provides the brief ideas of IR algorithm; and Sec. 2.5 details the phantoms used in this study and the process of data generation and acquisition. Results of dose reduction based on the comparison between algorithms are then shown in Sec. 3 followed by conclusions and discussions in Sec. 4.

## 2 Materials and Methods

### 2.1 Scanning Linear Observer

In this study, we focus on the application of the CSLO on CT imaging systems. The concept of scanning linear estimation^14^ is to approximate the mode of the posterior density and perform a pseudo maximum a posteriori (MAP) estimation. This requires a scan of parameter space to compare solutions and find the maximum. The general formula of MAP estimation is

θ^MAP=argmaxθ[pr(θ|g)]=argmaxθ[pr(g|θ)pr(θ)pr(g)], (1)

where θ and θ^ are the parameters we are interested in and the estimated parameter, respectively. The probability density function pr(g|θ) is the likelihood of data conditioned on the parameters to be estimated. To easily optimize this function, we will consider the Gaussian likelihood. Note this approximation does not imply that joint pdf pr(g,θ) is also Gaussian. Based on this approximation, the conditional likelihood pr(g|θ) can be described by

$$\mathrm{pr}(g|\theta )≅\frac{1}{\sqrt{2\pi^{M}\det(K_{g|\theta })}}\exp\{\frac{-1}{2}{\left[g-\overset{¯}{g}(\theta )\right]}^{t}K_{g|\theta }^{-1}[g-\overset{¯}{g}(\theta )]\},$$ (2)

where $\overset{¯}{g}(\theta )$ is the mean image averaged over the parameters θ, $K_{g|\theta }$ is the sample covariance matrix conditioned on the parameters, and det(·) is the determinant of the matrix. Instead of evaluating the covariance matrix and its inverse for every parameter, our second approximation is to use the mean of $K_{g|\theta }$ averaged over all θ as was done in Ref. 14. To avoid the exponential term of Eq. (2), the common strategy is to operate the natural logarithm on both sides and ignore the term independent of the parameters θ, which leads to

$$\ln[\mathrm{pr}(g|\theta )]≅\frac{-1}{2}{\left[g-\overset{¯}{g}(\theta )\right]}^{t}{\overset{¯}{K}}_{g}^{-1}[g-\overset{¯}{g}(\theta )]+\ln[\mathrm{pr}(\theta )].$$ (3)

Thus, the scanning linear estimator that maximizes the posterior density under these approximations is equivalent to

θ^SL(g)=argmaxθ{g¯(θ)tK¯g−1g−12g¯(θ)tK¯g−1g¯(θ)+ln[pr(θ)]}. (4)

The first term is a linear operation applied on the testing image data by $\overset{¯}{g}{\left(\theta \right)}^{t}{\overset{¯}{K}}_{g}^{-1}$ from the training data set. The second term is a shifted term due to the different parameters θ. The third term pr(θ) is assumed to be a flat prior, where all values of θ are equally likely. Thus, this third term is a constant, independent of θ, and ignored. So, the final equation becomes

θ^SL(g)=argmaxθ[g¯(θ)tK¯g−1g−12(θ)tK¯g−1g¯(θ)]. (5)

This observer operates on the data linearly, even though, in general, the linear template is a nonlinear function of θ. In the estimation process, the observer seeks the value of θ that will maximize this linear operation and give the estimated parameter θ^SL,θmax.

### 2.2 Channel Selection and Channelization

As mentioned earlier, we chose to use the CSLO to perform our combined detection/estimation tasks. This observer model has the benefit of being computationally practical and also relevant in terms of approximating human-observer performance. Many observer models, including the CSLO, require the estimation and inversion of a covariance matrix. The size of the regions of interest (ROI) patches in our study is M=100×100  pixels. The minimum number of sample images to achieve an invertible estimate of an M×M covariance matrix is M+1. To make the inverse of a covariance matrix practical, we reduce the dimension of dataset through channelization. The channels selected in our study are 10 dense-difference of Gauss (DDOG) channels.^15^^,^^16^ The 10 DDOG channels employed in this study have been demonstrated to match the human-observer behavior in signal-detection tasks^16^ for radially symmetric signals. In addition to reducing the dimension of the covariance matrix, DDOG channels also have been proved to have a property of mimicking human visual system^15^^,^^16^ in the pure detection task. These channels have not been verified to match human-observer performance when used in a scanning observer as we are using them in this work. However, we chose to use this observer model, because the channels are based on the human visual system, even if the scanning mechanism is not.

The channelization process maps the image onto the channels. This process can be expressed as

$$g^{\mathrm{ch}}=T^{t}g,$$ (6)

where the superscript ch and t refers to channelized data and transpose, respectively, and T is the channels. The dimension of g is M, which, for this study, is $100^{2}$. The dimension of $g^{\mathrm{ch}}$ is 10 for the 10 DDOG channels. Thus, the minimum number of training images to achieve an invertible estimate of the covariance matrix is 11. The channelized image data $g^{\mathrm{ch}}$ is used as input in Eq. (5), and the final equation for CSLO in this study becomes

θ^CSL(g)=argmaxθ[g¯2ch,training(θ)TK¯g¯2ch,training−1gch,training−12g¯2ch,training(θ)TK¯g¯2ch,training−1g¯2ch,training(θ)], (7)

where ${\overset{¯}{g}}_{2}^{\mathrm{ch},\text{training}}(\theta )$ is the average of signal-present image from training image dataset [Eq. (8)], ${\overset{¯}{K}}_{{\overset{¯}{g}}_{2}^{\mathrm{ch},\text{training}}}^{-1}$ is the covariance matrix calculated from ${\overset{¯}{g}}_{2}^{\mathrm{ch},\text{training}}(\theta )$ and averaged over different signal parameter vectors θ [Eqs. (9) and (10)], and $\;g^{\mathrm{ch},\text{testing}}$ is either a signal-present or signal-absent image. The second term in Eq. (7) is independent of testing data, but it has to be retained since it is θ dependent.

### 2.3 Tasks and Test Statistics

The CSLO was designed for pure estimation tasks but can be readily extended to include detection by including the value of the maximum in Eq. (7) as the test statistic. Thus, the test statistic that is used to decide whether the signal will be called present or not is the maximum of Eq. (7) instead of the argument maximum. This is similar to the scanning Hotelling observer.^17^^,^^18^ Note here that if $\;g^{\mathrm{ch},\mathrm{testing}}$ in Eq. (7) is a signal-present image, then the test statistic generates a random variable as $t_{\mathrm{CSL},2}$; on the other hand, if $\;g^{\mathrm{ch},\mathrm{testing}}$ is from signal-absent image pool, then the test statistic generates a random variable $t_{\mathrm{CSL},1}$. A decision that the signal is present is made when the test statistic is greater than the test-statistics threshold; otherwise, the observer decides that the signal is absent. For combination tasks that include detection and estimation, the CSLO scans parameters in the parameter domain to search for θ that maximizes Eq. (7). θ is a parameter vector including locations, sizes, and contrasts of signals. When the estimated parameters θ^CSL, the output of Eq. (7), are equal to the true parameters $\theta_{\text{true}}$, the estimation is counted as being correct.

**Fig. 1:**
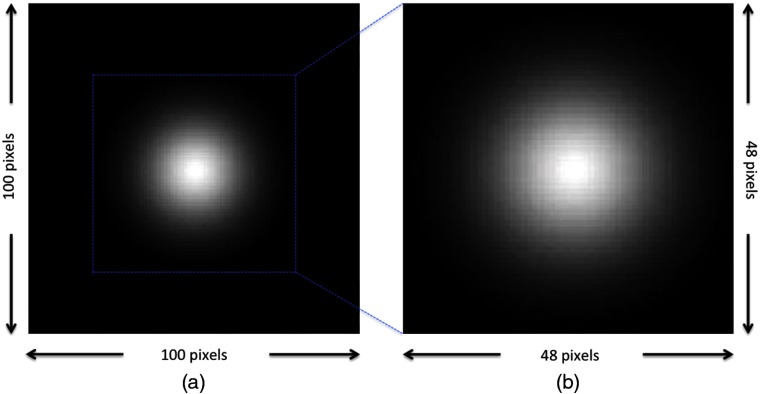
Templates: (a) original EROC template and (b) cropped EROC template. The cropped EROC template is used for scanning on the spatial domain for signal-location-unknown tasks.

**Fig. 2:**
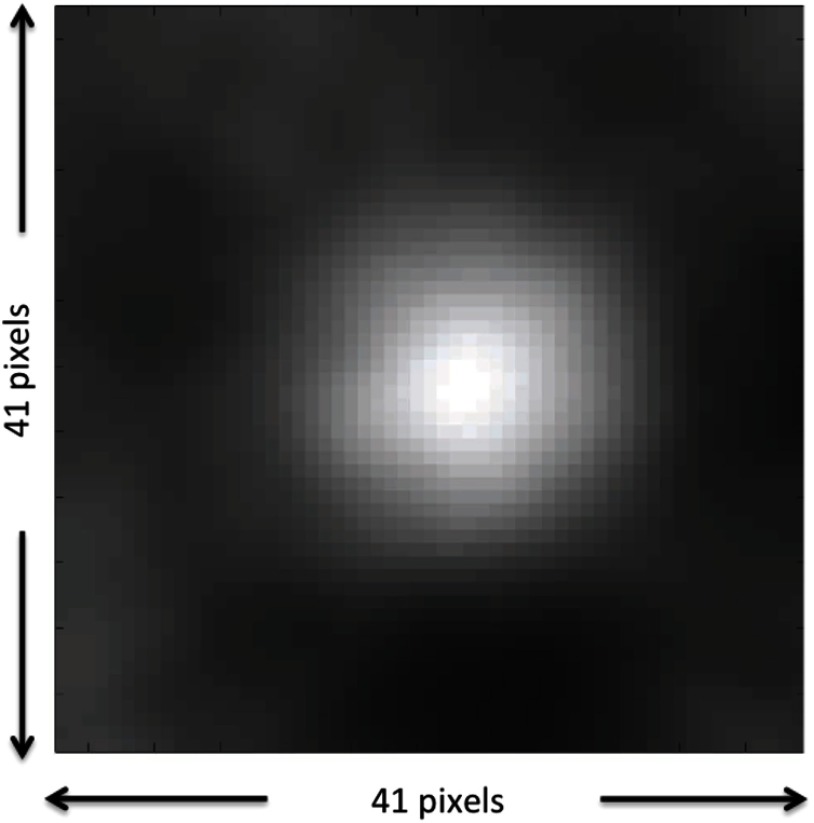
Example of a test statistic map. After cropped EROC template of one specific signal scanned on all possible locations of a signal on a testing image, a statistic map associated to this testing signal is generated. The highest pixel value (brightest spot) in this map indicates the estimated location of the testing signal.

Note that the implementation of Eq. (7) is potentially nonlinear in θ but is linear in the image data. Thus, we refer to the implementation of Eq. (7) as an EROC template where the template itself depends on the parameters to be estimated (e.g., location, size, and contrast). To implement location estimation, we crop the EROC templates down (Fig. 1) to exclude the mostly zero portions of the template. This increases computational efficiency substantially. The cropped EROC template contains all of the information of original image template but with smaller size. With this approximation, a cropped EROC template was scanned at every possible location and for every possible value of size and contrast on every testing image. There were 41×41 possible locations, 5 possible sizes, and 4 possible contrasts in the signal-present images. A statistics map (Fig. 2) was generated after the cropped EROC template was scanned over a single testing image. If the test statistic is above threshold, the observer model then chooses the location of signal based on the highest pixel value in the statistics map and estimates the size and contrast for the given signal in the testing image based on the value of θ. For signal-present images, if the difference between the estimated location and the true location (the center) was less than location threshold (the radius of the smallest signal) and the estimated size and contrast were equal to the true values, this combined detection and estimation was considered successful. According to the test-statistics thresholds, two test statistic distributions ($t_{\mathrm{CSL},\;2}$, $t_{\mathrm{CSL},\;1}$) and the results of estimation, an estimation receiver operating characteristic (EROC) curve^19^ can be generated. The area under the EROC curve (EAUC) value is the figure of merit used in this study. The higher the EAUC value, the better the image quality of the system under the combination of detection and estimation tasks.

### 2.4 Training, Testing, and Variance Estimation

The training data are used to estimate the parameters that define the model observers, i.e., the means and the covariance matrices. As mentioned before, although the minimum required training samples’ size is 11, to have more accurate estimate statistics, 200 samples were used for training and 300 samples were used for testing. For the CSLO, the training dataset is used to estimate ${\overset{¯}{g}}_{2}^{\mathrm{ch},\text{training}}(\theta )$, and ${\overset{¯}{K}}_{{\overset{¯}{g}}_{2}^{\mathrm{ch},\text{training}}}$, and the equations are given by

$${\overset{¯}{g}}_{2}^{\mathrm{ch},\text{training}}(\theta )=\frac{1}{N}\sum_{i=1}^{N}g_{2,i}^{\mathrm{ch},\text{training}}(\theta ),$$ (8)

$$K_{{\overset{¯}{g}}_{2}^{\mathrm{ch},\text{training}}}(\theta )=\frac{1}{N}\sum_{I=1}^{N}[g_{2,i}^{\mathrm{ch},\;\mathrm{training}}(\theta )-{\overset{¯}{g}}_{2,}^{\mathrm{ch},\mathrm{training}}(\theta )]\times {\left[g_{2,i}^{\mathrm{ch},\mathrm{training}}(\theta )-{\overset{¯}{g}}_{2}^{\mathrm{ch},\mathrm{training}}(\theta )\right]}^{t},$$ (9)

$${\overset{¯}{K}}_{{\overset{¯}{g}}_{2}^{\mathrm{ch},\text{training}}}=\frac{1}{\text{number of}\;\theta \;}\sum_{\theta }K_{{\overset{¯}{g}}_{2}^{\mathrm{ch},\text{training}}}(\theta ).$$ (10)

Once these parameters were estimated, the testing images were substituted into Eq. (7) to estimate the EAUC values.

There are several ways to estimate the variance of the EAUC: bootstrap,^20^ jackknife,^21^ and shuffle^22^ methods. They all have been studied previously.^23^^–^^28^ In this work, the variance of EAUC values is estimated by a completely nonparametric and unbiased approach, referred to as the Barrett–Clarkson–Kupinski method or the one-shot method.^29^ This variance-estimation method is a variant of the original Dorfman–Berbaum–Metz technique^30^^–^^32^ but does not rely on resampling techniques.

### 2.5 Computed Tomography Iterative Reconstruction Approach Studied in this Work

To achieve good image quality at low flux CT imaging conditions and maintain reconstruction speeds comparable to FBP, we have designed an IR approach that de-emphasizes the system optics modeling. Imaging-chain statistical noise modeling, physics modeling, and object modeling have been included in this IR algorithm. The detailed description of this algorithm will be presented in a future publication. The focus in this paper is to apply the model observer approach designed above to quantitatively evaluate the dose saving or image quality improvement capability of this IR algorithm developed in house.

### 2.6 Phantoms, Data Acquisition, and Generation

**Fig. 3:**
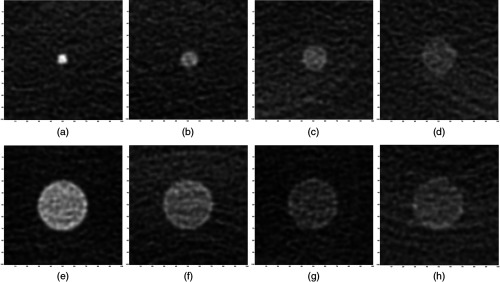
Examples of images used for combination of detection, size and contrast estimation task. All of the images shown are the averaged results of 500 FBP images. The various signal parameters are: (a) 3 mm 14 HU, (b) 5 mm 7 HU, (c) 7 mm 5 HU, (d) 10 mm 3 HU, (e) 15 mm 14 HU, (f) 15 mm 7 HU, (g) 15 mm 5 HU, and (h) 15 mm 3 HU.

In this work, low contrast (LC) objects embedded in the Medical Imaging Technology Alliance (MITA) CCT 189 phantom (Medical Imaging and Technology Alliance constructed by the Phantom Laboratory) were imaged on a GE Discovery CT750 HD CT system. Examples of signal-present images are shown in Fig. 3. In this study, the MITA phantom was used for signal detection and signal size and contrast estimation task. The properties of LC objects in this phantom are listed in Table 1. Only axial scans were used in this work. The large body bowtie was used for the MITA CT IQ LCD phantom with the body ring attached. The slice thickness was 0.625 mm, and the collimator aperture used was 20 mm.

**Table 1:** MITA body phantom signal parameters.

Object	Diameter (mm)	Contrast relative to background (HU)
1	3	14
2	5	7
3	7	5
4	10	3
5	15	14
6	15	7
7	15	5
8	15	3

In this study, the x-ray tube current was varied to achieve different radiation dose levels. For each dose level, 50 identical scans were acquired. A total of 10 signal-present and signal-absent ROI pairs were extracted from different longitudinal locations (along the CT system table direction) from each scan. The 50 scans and 10 extracted ROI pairs per scan resulted in 500 individual ROI pairs for each LC object at every dose level. Images were reconstructed at a field of view 180 mm with a matrix size of 512×512 image pixels. A random order of 500 images was split into training and testing image datasets. The same randomized sequence was used for every study. The center of each signal was determined by analyzing the mean image. The signal was always in the center of the ROI, and the signal-absent ROIs were extracted from regions distant from the signals to avoid any overlap between signal-present and signal-absent ROIs.

For each object and dose level selected, there were 500 independent signal-present and signal-absent image pairs. These image pairs were randomly split into 200 pairs for training and 300 pairs for testing in the observer study. EROC curves for the combined detection and estimation task were generated, and the corresponding areas under the curves were calculated. As mentioned above, the variances of EAUC values were estimated via the one-shot method.^29^

## 3 Results

**Fig. 4:**
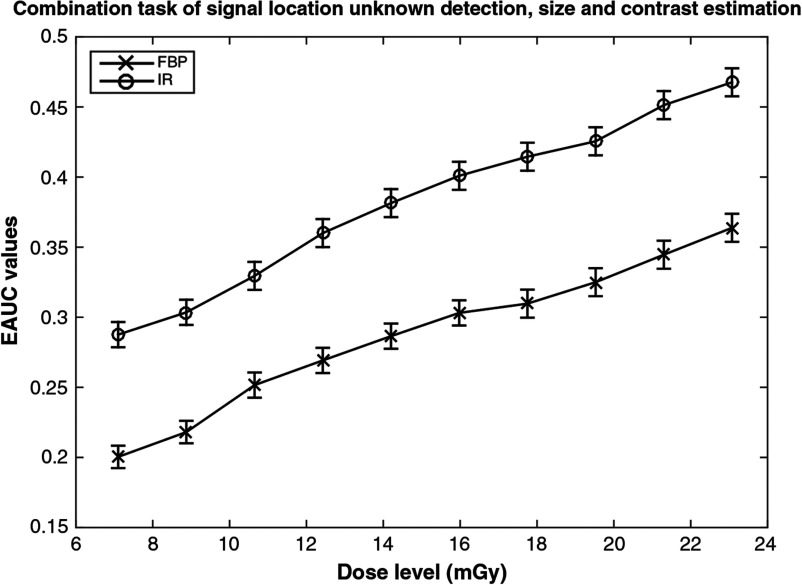
An example of QDC curve for the task of signal detection, localization, and estimation of size and contrast.

FBP and IR were compared for different tasks. Quality-dose characteristic (QDC) curves^33^ (Fig. 4) were generated by plotting different dose levels and their associated EAUC values for both FBP and IR algorithms. For each task, 50 scans were made for each dose level and there were 10 different dose levels as shown in Fig. 4. The results show that the QDC curve of IR is higher than that of FBP at all dose levels. It suggests that the IR is better than FBP in terms of image quality at the same dose level. A comparison could also be made across dose levels for an equal EAUC value. This comparison indicates that the required dose level is lower for IR than FBP to achieve the same image quality. Based on these QDC curves, achievable dose reduction can be easily derived. All objects in the MITA phantom were scanned axially for all experiments using the parameters mentioned above, and signal-location-unknown detection, size, and contrast estimation were performed.

**Fig. 5:**
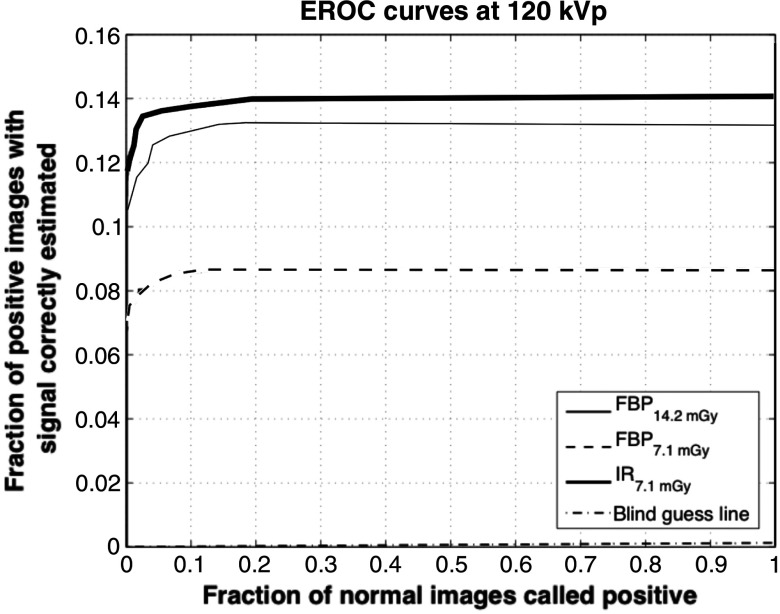
EROC curves (MITA body phantom). The combination of signal-location-unknown detection, size, and contrast estimation task.

**Fig. 6:**
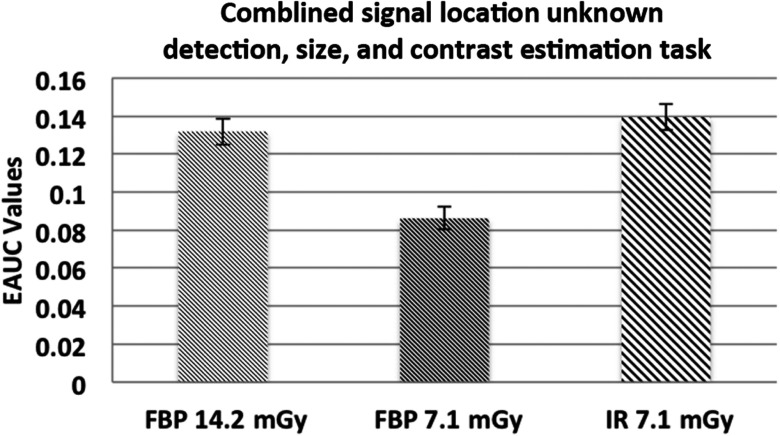
EAUC values and associated error bars of the combined task of detection.

Because the location of signal is unknown for the observer, the observer had to localize the signal in the provided testing image first and followed by estimating the size and the contrast of the signal. As mentioned in Sec. 2, the location threshold is about the radius of the smallest signal. The total number of possible locations for signals is 41×41. Thus, similar to signal location known study, the guessing value of EAUC is given by

$$\frac{1}{2}\frac{\text{the area of a circle of radius equal to the location threshold}}{\text{the number of objects}\times \text{the number of possible locations}},$$ (11)

which, for this problem, is 0.0021. The result indicates that a 50% dose reduction could be achieved using the IR algorithm. EROC curves are shown in Fig. 5. The significant difference (p<0.01) between the EAUC value of IR low dose and FBP low dose is shown in Fig. 6.

The improvement of image quality of IR is substantial. This improvement not only helps observers to determine if tumors exist and where the tumors located relatively correctly but also helps observers to distinguish the physical properties such as the size and the contrast of the targeted lesion easily.

## 4 Discussions and Conclusions

Different IR algorithms have been designed on CT systems to assist the patient radiation dose reduction. The nonlinearity nature of both the CT system and an IR algorithm makes the assumptions of a linear and shift-invariant system far from valid. Thus, conventional image-quality metrics such as modulation transfer function, noise power spectrum, and detective quantum efficiency while well-suited for characterizing detector performance, are not well-suited for overall CT image quality evaluation. In view of this, mathematical model observers have been selected as surrogates to study the task-based image quality performance of CT imaging systems. In this work, we used CSLO to measure the image quality under a more realistic task, the detection, and estimation, at different dose levels for two different reconstruction methods. Radiation dose levels required for LC object detection and estimation in body phantoms were examined and evaluated quantitatively. This work provides an objective way to assess the image quality of CT imaging systems for dose saving evaluation. However, unlike pure detection tasks, currently there is no proper way of adding internal noise in the combination of detection and estimation tasks so internal noise was not used in this study. Our results provide the upper bounds of observer studies.

In this study, different combinations of clinically relevant tasks were considered and evaluated by CSLO. Our results suggest that CSLO could be used as an image quality assessment tool for performance evaluation of CT imaging systems. Future works include the addition of internal noise, correlation with human-observer performance, and head mode phantom evaluations. In addition, we note that conclusions drawn from this study must be tempered by the fact that the phantom is uniform. Thus, adding anatomical variability, possibly through the use of real patient data, will be a possible topic in the future. Additionally, having more signals of different sizes and contrasts in the new design of phantom is in the future plan as well. On the other hand, this method currently is still time-consuming and requires numerous images. To make this method more practical, further design of the CSLO using fewer images is needed and work has progressed in this area.^34^ Much like the differences in dose savings we observed when the tasks were made more complex, we expect that there will be differences in the performance when anatomical variability and more complicated signal models are used.

In this study, we evaluated the performance of FBP and an IR algorithm. Our results indicate that the IR algorithm can make a significant dose reduction without compromising image quality. According to our results, the performance of the IR algorithm shows that the noise has been decreased greatly compared to the traditional FBP algorithm for all of the combinations of detection and estimation tasks. Approximately 50% dose reduction can be achieved using this IR algorithm for tasks that combine signal-location-unknown detection and estimation of signal size and contrast. Thus, to achieve the same image quality, the IR algorithm requires less x-ray radiation exposure to patients. To provide this benefit of lower radiation dose to patients, our results support the use of IR in clinical CT. Further study is needed with anatomical variability, real data, and even more complicated tasks to further validate this dose-reduction claim.
